# Feasibility and acceptability of two incentive-based implementation strategies for mental health therapists implementing cognitive-behavioral therapy: a pilot study to inform a randomized controlled trial

**DOI:** 10.1186/s13012-017-0684-7

**Published:** 2017-12-15

**Authors:** Rinad S. Beidas, Emily M. Becker-Haimes, Danielle R. Adams, Laura Skriner, Rebecca E. Stewart, Courtney Benjamin Wolk, Alison M. Buttenheim, Nathaniel J. Williams, Patricia Inacker, Elizabeth Richey, Steven C. Marcus

**Affiliations:** 1 Department of Psychiatry, University of Pennsylvania Perelman School of Medicine, 3535 Market Street, 3015, Philadelphia, PA 19104 USA; 2 School of Social Service Administration, University of Chicago, Chicago, USA; 3 New York-Presbyterian Hospital, Weill Cornell School of Medicine, New York, USA; 4 School of Nursing, University of Pennsylvania, Philadelphia, USA; 5 The Center for Health Incentives and Behavioral Economics, University of Pennsylvania, Philadelphia, USA; 6 School of Social Work, Boise State University, Boise, USA; 7 Hall-Mercer Community Mental Health Center, Philadelphia, USA; 8 The Village, Philadelphia, USA; 9 School of Social Policy and Practice, University of Pennsylvania, Philadelphia, USA

**Keywords:** Evidence-based practices, Incentives, Behavioral economics, Community mental health

## Abstract

**Background:**

Informed by our prior work indicating that therapists do not feel recognized or rewarded for implementation of evidence-based practices, we tested the feasibility and acceptability of two incentive-based implementation strategies that seek to improve therapist adherence to cognitive-behavioral therapy for youth, an evidence-based practice.

**Methods:**

This study was conducted over 6 weeks in two community mental health agencies with therapists (*n* = 11) and leaders (*n* = 4). Therapists were randomized to receive either a financial or social incentive if they achieved a predetermined criterion on adherence to cognitive-behavioral therapy. In the first intervention period (block 1; 2 weeks), therapists received the reward they were initially randomized to if they achieved criterion. In the second intervention period (block 2; 2 weeks), therapists received both rewards if they achieved criterion. Therapists recorded 41 sessions across 15 unique clients over the project period. Primary outcomes included feasibility and acceptability. Feasibility was assessed quantitatively. Fifteen semi-structured interviews were conducted with therapists and leaders to assess acceptability. Difference in therapist adherence by condition was examined as an exploratory outcome. Adherence ratings were ascertained using an established and validated observational coding system of cognitive-behavioral therapy.

**Results:**

Both implementation strategies were feasible and acceptable—however, modifications to study design for the larger trial will be necessary based on participant feedback. With respect to our exploratory analysis, we found a trend suggesting the financial reward may have had a more robust effect on therapist adherence than the social reward.

**Conclusions:**

Incentive-based implementation strategies can be feasibly administered in community mental health agencies with good acceptability, although iterative pilot work is essential. Larger, fully powered trials are needed to compare the effectiveness of implementation strategies to incentivize and enhance therapists’ adherence to evidence-based practices such as cognitive-behavioral therapy.

**Electronic supplementary material:**

The online version of this article (10.1186/s13012-017-0684-7) contains supplementary material, which is available to authorized users.

## Background

The importance of implementing evidence-based practice (EBP) in community mental health agencies has been well established [1], yet EBP is not widely used in these settings [2]. Use of evidence-based psychosocial treatments that have been systematically and rigorously evaluated [3] in public mental health systems results in better therapeutic outcomes and a cost-benefit advantage over treatment as usual [4]. In recent years, policy makers have invested resources to develop infrastructure to support EBP in many large mental health systems [1]; yet the integration of EBP into practice continues to be highly variable [5, 6].

There is a growing interest in identifying effective implementation strategies that will increase adoption, implementation, and sustainment of EBP. However, most prior implementation studies in community mental health settings have focused on training and consultation, despite evidence that these strategies do not produce sustainable changes in therapist behavior [7, 8]. This pilot study seeks to understand the feasibility and acceptability [9] of two incentive-based implementation strategies that have been used in health settings and have direct relevance to and implications for policy and practice in public mental health systems across the USA.

One of the most robust findings from our efforts to study EBP implementation over the past 5 years in the Philadelphia public mental health system is that therapists do not feel rewarded or recognized for using EBP, thus potentially limiting their motivation to incorporate these interventions into their practice [5, 10–12]. Recent work in healthcare has used incentive-based strategies to change provider and patient behavior, drawing from behavioral economics, which offers insights for manipulating incentive design to account for the psychological biases that drive human behavior [13]. These strategies have shown robust effects in behavior change for both patients and providers [14–18]. Two incentive-based implementation strategies that could be deployed in mental health include financial and social incentives [19]. Incentives and rewards fall under the “reward and threat” category within the Behavior Change Technique Taxonomy. Specifically, incentives refer to informing individuals that the delivery of money, vouchers, or valued objects will be delivered if there is an effort to perform a behavior. Rewards refer to giving an individual money, vouchers, or valued objects when an effort to perform a behavior occurs [20].

Financial incentives assume that variation in clinician performance is caused by variability in motivation and that financial incentives will add to motivation [21]. Broadly across healthcare practices, a Cochrane Report systematic review found that financial incentives may be effective in changing healthcare professional practice, particularly when improving processes of care [22]. A few studies have examined the effectiveness of financial incentives for implementation of substance abuse interventions in drug and alcohol settings [23, 24] and suggest that financial incentives may be a powerful lever to change substance abuse counselor behavior in the short term [23]. More recently, the National Child Traumatic Stress Network called for the use of financial incentives in the delivery of trauma-focused cognitive-behavioral therapy (TF-CBT) nationally [25], although such incentives for delivery of EBP have not been studied.

An alternative strategy, social incentives, also assumes that variation in performance is caused by variability in motivation, but that the basis of this motivation is a desire to uphold personal, internalized professional standards and a high level of quality care. When public recognition of a peer reveals discrepancies between an individual’s behavior and that of her peers, particularly in the presence of strong professional norms, she is motivated to reduce the discrepancy by changing her behavior. This activation of peer comparisons [19] can be particularly salient for professions that have strong norms such as mental health providers. Leveraging social incentives to change therapist behavior, such as having a supervisor or leader of an agency publicly recognize therapist performance, has been lauded as a potentially effective strategy in the healthcare literature [19, 26, 27]. However, while early research suggests this approach is promising [28], social incentives have not been studied in mental health.

The primary aims of this pilot study were to evaluate the feasibility and acceptability of two incentive-based implementation strategies in mental health settings. An exploratory aim was to examine the preliminary comparative effectiveness of the implementation strategies on therapist adherence to cognitive-behavioral therapy (CBT), an EBP. Pilot results from two community mental health agencies will inform the design of a large cluster randomized trial that will be powered to test the effectiveness of the implementation strategies on adherence to CBT. Specifically, we hypothesized that the two implementation strategies would be feasible and acceptable in community mental health agencies from the perspectives of therapists and leaders.

## Methods

This study served as a pilot study to inform the design of a larger trial. Data from this pilot study will not be included in the main study.

### Setting

This study took place in the child outpatient programs of two community mental health agencies that were actively implementing CBT as part of a larger implementation initiative in the city of Philadelphia [29]. We elected to approach two agencies that were representative of the landscape of providers in the city of Philadelphia based on the number of therapists employed and youth served annually. Our goal was to recruit approximately ten therapists so that we could ascertain the feasibility and acceptability of the study procedures, as consistent with the goals of a pilot study.

### Participants

#### Therapist participants

**Fig. 1:**
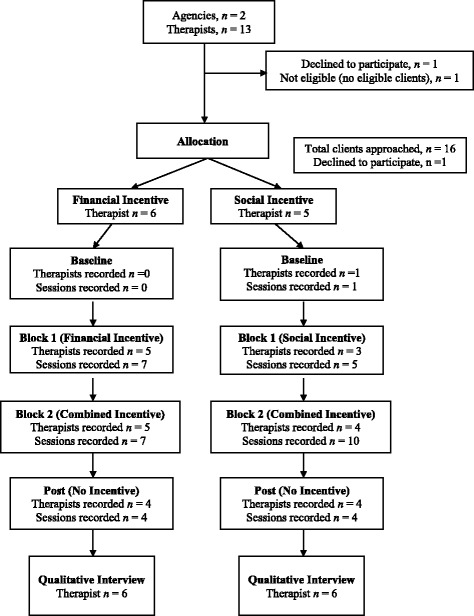
Flow chart of the inclusion and allocation of agencies and participants

Therapists (*n* = 11) were predominantly female (*n* = 9, 81.8%) and were an average of 37.1 years old (*SD* = 10.6). Therapists identified as White (*n* = 7, 63.6%), Hispanic/Latino (*n* = 4, 36.4%), and Black (*n* = 1, 9.1%). All therapists held master’s degrees and had an average of 6.64 years of full-time clinical experience (*SD* = 5.26). See Fig. 1.

#### Client participants

Clients (*n =* 15) averaged 12.8 years of age (*SD* = 4.13) and were predominantly Black (*n* = 13, 86.7%). One client identified as Hispanic/Latino and another identified as American Indian. The primary diagnosis of most clients as reported by their therapist was post-traumatic stress disorder (*n* = 10, 66.7%); three clients had adjustment disorder, and two had depressive disorder diagnoses. The average number of completed sessions at the time that clients were first enrolled in the study was 10.73 sessions (*SD* = 5.62).

#### Leader participants

Leaders (*n* = 4) were all female and identified as White. Leaders included clinic directors and direct supervisors, were an average of 45.3 years old (*SD* = 15.42), and had an average of 20.50 years of full-time clinical experience (*SD* = 16.05); all held master’s degrees.

#### Study design

**Fig. 2:**
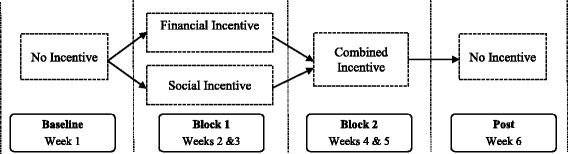
Overview of study design

We collected data at each agency for 6 weeks (see Fig. 2). In week 1, therapists were not eligible to receive an incentive nor feedback report (i.e., baseline). In weeks 2–3 (block 1), therapists were randomized to receive either the financial incentive (FI) *or* social incentive (SI). Six therapists were randomized to FI, and five therapists were randomized to SI. In weeks 4–5 (block 2), therapists were eligible to receive both FI *and* SI (i.e., a dual incentive). One audio-recorded client session within each block was randomly selected per therapist; therapists received the reward if this randomly selected session met the criterion for adherence to CBT (described below). In week 6 (i.e., post-incentive), therapists were not eligible to receive an incentive nor feedback report but provided recordings of their in-session behavior.

#### Procedure

We reached out to agency leaders to ascertain their interest in participation. Subsequently, we met with each leader to describe the study and obtain their feedback on its design. To launch the study, we held a 1-h meeting with all eligible therapists, described the incentives in depth, and ensured that participants learned the criteria that would be used to determine incentive eligibility. Therapists were aware of the behaviors that they needed to engage within to receive the incentive prior to engaging in the interventional component of the study (blocks 1 and 2). During this meeting, therapists were told that they would be randomly assigned to FI *or* SI during block 1 of the study and that they would be eligible for both FI *and* SI during block 2 of the study. This approach was selected at the request of agency leaders who wanted all therapists to have the opportunity to earn the financial incentive at some point in the study. Participants were randomly assigned using simple randomization procedures to one of the two conditions. The randomization sequence was generated by the principal investigator with a 1:1 allocation. Conditions were written on a folded piece of paper that therapists selected out of a hat (i.e., therapists and research staff were not able to see which condition the therapist selected until they opened the paper). The research staff and therapists were not blind to conditions. A trained research assistant was assigned to each agency to facilitate patient recruitment. All clients between the ages of 7–24 of enrolled therapists receiving individual face-to-face CBT at one of the two agencies participating were eligible for the study. Study participation for clients entailed granting permission for their session to be recorded. Both the guardian (if the client was under 18) and youth had to consent and assent to have the session recorded. The youth were paid $10 dollars for each session that was recorded.

Following study completion, all participating therapists and leaders in the two agencies completed individual qualitative interviews with trained research assistants that queried about the acceptability and feasibility of the conditions, study design, and research procedures. Data collection was completed from September to December in 2015. The pilot trial ended when data completion was complete at each agency.

### Clinical intervention

The clinical intervention of interest is CBT. CBT has amassed a large body of evidence supporting its effectiveness as a treatment for a wide range of youth psychiatric disorders [30–32]. CBT refers to a group of interventions that share the underpinning that psychiatric disorders are maintained by cognitive and behavioral factors. These interventions target maladaptive cognitions and behaviors to result in symptom reduction and improved functioning [30]. Common strategies used in CBT include managing negative thoughts (e.g., cognitive restructuring), changing maladaptive behaviors (e.g., exposure), managing maladaptive mood and arousal (e.g., behavioral activation), and general skill training (e.g., problem-solving; [33]).

### Implementation strategies

The financial incentive condition was designed based on principles of behavioral economics [19, 21]. Specifically, financial rewards were paid separately from regular salary to increase saliency [14, 34] and were delivered quickly following desired behavior [19]. Therapists were randomized to earn $100 if a randomly selected session over the 2-week period met criterion for adherent CBT delivery; the reward was provided in cash within 1 week. The social incentive was designed to activate peer comparisons via public recognition within the therapist’s agency. We designed the social incentive condition such that therapists would receive public recognition if a randomly selected session over the 2-week period met criterion for adherent CBT delivery. Specifically, their leader sent out an email to the entire team celebrating that the therapist met the target behavior (i.e., social reward). The email (see Additional file 1) was drafted by the research team to ensure consistency.

Based upon initial conversations from the leaders and therapists, we also provided therapists in both arms with a feedback report for the randomly selected session from each block of study participation (i.e., weeks 2–3; 4–5). These one-page feedback reports were written by doctoral-level clinical psychologists and described the areas of strength and opportunities for improvement in therapist delivery of CBT.

#### Target behavior for rewards

The target behavior for receipt of reward was therapist adherence to CBT. To determine if therapists were implementing CBT in an adherent manner, we used the *Therapy Process Observational Coding System for Child Psychotherapy-Revised Strategies Scale* (TPOCS-RS) [35]. The TPOCS-RS is the gold-standard observational coding system designed to capture the extensiveness of adherence to a range of psychosocial interventions for youth, including CBT, and shows good internal consistency and validity [35, 36]. The tape from every recorded session (including the one randomly selected for evaluation) was assessed by a doctoral-level rater trained in the TPOCS-RS by the instrument developer. Training followed established procedures [35]. Prior to initiating coding in this study, the rater independently coded 30 certification sessions and calculated intraclass correlation coefficients (ICCs) against gold-standard ratings. The rater exceeded the TPOCS-RS certification standard of ICCs > .59 for all items (ICC range = .76–.97).

We focused on the TPOCS-RS CBT subscale that includes 12 CBT interventions (see Table 1 for interventions). Each component was rated from 1 (*not at all*) to 7 (*extensively*). While the TPOCS-RS has several options for scoring (see [37] for discussion), we used the maximum score approach, in which the highest score across the 12 intervention components is used. We based our decision to use the maximum score approach because we would not expect a therapist to use all 12 components in each session. Delivering any CBT model component with extensive adherence is the goal behavior. For example, a therapist might use *cognitive education* (extensiveness = 7) and *relaxation* (extensiveness = 3), but not use *psychoeducation* (extensiveness = 1) or *exposure* (extensiveness = 1). The therapist would receive a 7 for that session. Thus, therapists could earn an overall maximum score of 1–7 for each therapy session they delivered. To be eligible for the reward, therapists’ maximum CBT score had to be a 4 or greater. We selected this as a benchmark based upon the empirical literature demonstrating that the average extensiveness scores obtained in therapists trained in CBT in usual care are 4 out of 7 [37]. During the initial recruitment meeting, we presented this information in detail so that therapists were aware of the scoring system and benchmark.

**Table 1:** Number of times each CBT intervention was scored

TPOCS-RS item	Times scored (% of sample)	Average rating when scored *M*, (*SD*; range)
Any intervention scored	37 (100)	
Psychoeducation	21 (56.8)	2.57 (.81; 2–4)
Cognitive education	22 (59.5)	3.00 (1.54; 2–7)
Cognitive distortion	10 (27.0)	2.40 (.97; 2–5)
Functional analysis	4 (10.8)	2.00 (0.0; 2–2)
Relaxation strategies	17 (45.9)	3.88 (1.80; 2–7)
Respondent strategies	4 (10.8)	5.75 (1.26; 4–7)
Behavioral activation	0 (0)	N/A
Coping skills	7 (18.9)	2.71 (1.50; 2–6)
Skill building	7 (18.9)	2.00 (0.0; 2–2)
Operant strategies (child)	19 (51.4)	2.21 (.42; 2–3)
Operant strategies (parent)	3 (8.1)	3.00 (1.00; 2–4)
Parenting skills	2 (5.4)	2.50 (.71; 2–3)

*TPOCS-RS* Therapy Process Observational Coding System for Child Psychotherapy-Revised Strategies Scale. Percentages reflect the number of sessions in which the intervention was coded across all sessions (*n* = 37)

*N/A* not applicable

### Analytic plan

We were interested in two primary outcomes (i.e., feasibility and acceptability) and an exploratory outcome (adherence to CBT). Feasibility and adherence were investigated quantitatively whereas acceptability was investigated using qualitative methods.

#### Feasibility

To ascertain feasibility, we calculated the ratio of (a) number of therapists agreeing to participate divided by number of eligible therapists in each program and (b) number of clients agreeing to be recorded divided by number of eligible clients approached to participate.

#### Acceptability

All qualitative interviews with therapists and leaders were audiotaped and transcribed verbatim. Transcripts were analyzed in an iterative process based upon an integrated approach that incorporated both inductive and deductive features [38]. Through a close reading of four transcripts, the investigators developed a set of codes that were applied to the data (i.e., inductive approach). A priori codes derived from the original research questions and previous literature were also applied (i.e., deductive approach). Specifically, acceptability, or how palatable or satisfactory participants found the implementation strategies to be, was of interest. A random subset of transcripts (20%) was coded by two investigators, and inter-rater reliability was found to be excellent (*ĸ* = .94) [39]. Through the approach described above, the first author produced memos including examples and commentary regarding themes that emerged from the codes to interpret the data [38].

#### Adherence

First, we examined if rates of reward receipt (i.e., the randomly selected tape received a score of 4 or higher on at least one TPOCS-RS item) differed between clients of therapists initially randomized to financial incentive (FI; *n* = 6) or social incentive (SI; *n* = 5) using Pearson chi-square tests. Second, we compared CBT adherence rates by initial randomization status across the full sample of sessions. Third, we compared CBT adherence rates between the active incentive project phase (i.e., blocks 1 and 2) and the post-period for each intervention arm.

### Ethics

All procedures were approved by the city of Philadelphia and University of Pennsylvania institutional review boards. Leaders within each community mental health agency were informed and gave permission for the study to be conducted within their respective sites. All therapist, leader, and client participants completed written, informed consent prior to initiating study participation. All participants were informed that they could withdraw from the study at any time.

## Results

### Feasibility

#### Agency feasibility

We reached out to two leaders of two community mental health agencies in Philadelphia. Both agencies (100%) agreed to participate.

#### Therapist feasibility

Eleven out of the 13 (85%) eligible therapists agreed to participate in the study. One therapist declined participation because s/he did not have any clients eligible for the study. The second therapist declined participation because s/he was a part-time clinician (i.e., independent contractor) and did not feel comfortable recording patient sessions. Therapists recorded 41 sessions across 15 unique clients over the project period (*M* = 3.73 sessions per therapist, *SD* = 1.95); one therapist who was randomized did not record any sessions.

#### Client feasibility

Fifteen out of the 16 (94%) eligible clients agreed to participate in the study. One client declined to participate.

### Acceptability

#### Therapist and leader study participation

All participants, including therapist and leader participants, reported that they found their overall experience with the study to be highly acceptable. A number of participants described the work that they do as “thankless work,” and remarked that receiving a financial or social reward for their contributions was motivating. One therapist said, “I get paid very little for a lot of work, and a lot of hard and heavy work,” further reflecting that the reward she received made her feel appreciated and acknowledged in a way that she rarely experienced.

#### Acceptability of the financial incentive

The majority of therapists and leaders reported that they found the financial incentive condition to be acceptable. One therapist reported, “It was awesome having money. It was nice and instant, and handing you the money was pretty cool, you know it was Christmas time so it was awesome.” A number of therapists noted that receiving actual cash immediately was also motivating. At the same time, some participants also struggled with the ethics of receiving additional remuneration for activities that they perceived as being part of their daily job. This quote, which was echoed by a few individuals almost verbatim, illustrates this concern, “I think you should be delivering your best work whether you are incentivized or not.” One leader also reflected the tension around this ethical issue by saying, “The business part of me is saying yes, absolutely incentivize them. The ethical side of me is like, oh that’s a good question. And then the business side is like, shut up ethical side.”

#### Acceptability of social incentive

The majority of therapists and leaders also reported that the social incentive condition was acceptable, because “being recognized by your supervisor for the work that you’ve done is great.” One of the leaders reported that s/he and the therapists found the social incentive so appealing that they picked it up as a department and started sending out emails recognizing outstanding therapists after the pilot study. Additionally, the leader reported that the social incentive condition had the unintended consequence of making independent contractor therapists “feel part of the group” in a way that they had not felt previously. A leader in the other agency reported that social incentives were already a part of the fabric of their agency; thus, they did not have as much of an impact on the therapists. Both leaders reported that the content of the email felt inauthentic because they did not draft it themselves.

#### Client study participation

Six of the participants (five therapists and one leader) reported that youth clients and their legal guardians found study participation acceptable (the other participants did not comment on client acceptability). One unintended consequence of the study as reported by a therapist was that participating in the study anecdotally increased client engagement/attendance to sessions. Additionally, two therapists expressed surprise at how interested in participating in research their clients and guardians were.

#### Acceptability of the feedback report

The majority of therapists and leaders reported that the feedback reports provided to the therapists as part of study participation were a very important feature of the study. One leader noted, “I think what was the most surprising was the feedback report. Initially, the financial incentive was the most exciting, but I think what turned out to be more exciting was the feedback.” Participants reported that they seldom had the opportunity to receive feedback on their in-session behavior and that receiving brief feedback reports gave them an opportunity to improve their practice.

### Adherence

**Fig. 3:**
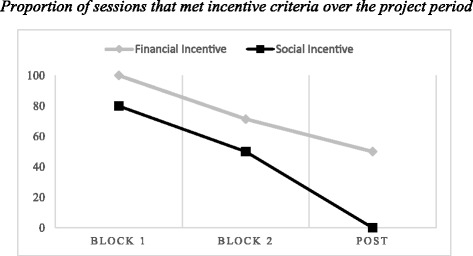
Figure displays proportions of sessions that met incentive criteria as a function of initial randomization status. In block 1 (weeks 2 and 3), therapists were randomized to receive either the financial or social incentive. In block 2 (weeks 4 and 5), therapists received both incentives. In post (week 6), therapists did not receive incentives

Table 1 shows the number of times each CBT intervention was scored using the TPOCS-RS items in this sample and the average rating when scored. The average maximum TPOCS-RS score across sessions was 4.08 (*SD* = 1.77; range = 2–7). First, we examined whether there were differences in adherence between therapists originally randomized to FI (*n* = 6 therapists) and those randomized to SI (*n* = 5 therapists) in block 1 only, using the intent to treat sample. A higher proportion of therapists’ randomized to FI received the reward (*n* = 5 of 6 therapists, 83.3%; 1 therapist did not recruit clients in block 1) relative to those randomized to SI (*n* = 2 of 4 therapists, 40%; two therapists did not recruit clients in block 1), although this was not significant (Fisher’s exact test *p* = .24). Initial randomization status was not associated with receiving the combined reward in block 2 (FI *n* = 5 of 6 therapists, 83.3%; SI *n* = 4 of 5 therapists, 80%; *X* ^*2*^ = .02, *p* = .89). Across all time points in the full sample of sessions (*n* = 37), 78.3% of CBT sessions delivered by therapists initially randomized to FI met criteria for the reward compared to 47.4% delivered by therapists initially randomized to SI (*X* ^*2*^ = 3.63, *p* = .06; see Fig. 3).

Next, we examined whether adherence was higher when therapists received either reward compared to the post-incentive period. Compared to the active incentive periods (Blocks 1 and 2 combined, *n* = 29 sessions across ten therapists), sessions recorded at post-incentive (total session *n* = 8 across eight therapists) were significantly less likely to meet the minimum threshold for the incentive (72.4% vs 25%, *X* ^*2*^ = 5.99, *p* = .01). When sessions recorded in the post-phase only were examined by initial randomization status, 2 out of 4 (50%) sessions delivered by therapists randomized to financial incentive met criteria for the incentive, and 0 out of 4 (0%) of public recognition sessions met criteria; this difference was not significant *(X* ^*2*^ = 2.67, *p* = .10).

## Discussion

The aims of this pilot study were to evaluate the feasibility and acceptability of two incentive-based implementation strategies, as well as to preliminarily explore the effectiveness of the implementation strategies on CBT adherence. This is consistent with the primary role of a pilot study which is to examine the feasibility of a research endeavor [9]. Findings suggest that the implementation strategies were both feasible and acceptable, but that some modifications will be necessary in a larger trial. Additionally, both implementation strategies we tested have the potential to change therapist behavior but financial incentives may be slightly more effective compared to social incentives.

Use of implementation strategies based upon principles of behavioral economics [40] was feasible, as indicated by the high recruitment rates at the agency (100%), therapist (85%), and client (94%) levels; which is notably higher than previous work conducted [5]. However, we faced challenges in the initial recruitment of clients, thus resulting in only one session recording during the baseline period. We learned from conversations with the participants that therapists would benefit from having time to introduce the study to youth in the week preceding recruitment. This was especially necessary given that adolescents typically came to session without their guardian, requiring advance notice to obtain caregiver consent. Future studies will include an extra week, prior to the baseline week, to facilitate client engagement either in person or by obtaining permission from the IRB to conduct verbal consent with guardians over the telephone as we did in a recent study [41].

We also gained information about the feasibility of the study design. In our early conversations with agency leaders, they shared their concern about randomization of therapist within agencies. They were wary about having half of their therapists in a financial incentive condition and the other half in a social incentive condition, given the less than optimal financial environment [12] in community mental health (i.e., they did not want therapists to feel resentful of their colleagues). Thus, we elected to use a design where all therapists had the opportunity to earn the financial incentive. Although this addressed the leader concerns, it also proved to be a complex design that was difficult to explain to study participants, limiting the feasibility of conducting a scaled-up version of this trial [42]. Future studies will randomize at the organization level so that providers receive a single implementation strategy, thus mitigating leader concerns.

In addition to positive indicators of feasibility, both the implementation strategies and participation in research were acceptable from the perspective of therapists and leaders. Corroborating principles from behavioral economics [21], therapists reported appreciating that they received the financial reward as cash (i.e., separate from their check to increase saliency) [14] as well as promptly, and around the holidays [19]. They generally reported finding both incentive types to be motivating, which is consistent with the underlying theory which supports the use of such strategies [21], and is particularly salient in public mental health settings where therapists are often underappreciated, overworked, and underpaid [10]. Interestingly, no therapists or leaders noted any negative implications of being subject to incentive strategies simultaneously within the context of the broader EBP implementation efforts taking place in their agencies [11, 29]. This may further support the feasibility of layering incentive-based strategies on top of standard implementation strategies (e.g., training and consultation), although this will need to be tested in future work. There are limitations to this pilot trial. First, we were unable to obtain baseline measures of behavior from therapists, thus limiting our ability to make conclusions about how the incentives changed their behavior. Second, we are unable to disentangle the effect of feedback reports alone on therapist behavior. Third, we did not randomly select the agencies we approached for this study, thus the findings may not be generalizable to other agencies.

There are future directions to consider regarding both financial and social incentives in community mental health settings. First, with regard to financial incentives, a number of participants raised concerns about the ethics of receiving additional remuneration for doing work that they perceived to be within the scope of their daily practice. These concerns have been raised in the larger healthcare literature (see [43, 44]), and there may be additional considerations for clinicians serving vulnerable populations such as youth with mental health difficulties. One particular area of interest is the potential impact of such incentives on the therapeutic alliance [45]. For example, client awareness that their therapists are receiving extra compensation for high-quality services may impact their willingness to trust their therapist; alternatively, therapists may use CBT with clients when it is not indicated in order to receive the incentive. However, if the incentives are designed in an ethnical manner with appropriate safeguards (i.e., maintaining freedom to make informed choices, minimize current healthcare inequities, and have rigorous monitoring and evaluation plans), then it is likely that the benefits outweigh the risks [44].

Another area of interest is the potential impact of financial incentives on clinicians’ intrinsic or internalized motivation to deliver high fidelity CBT [46]. Behavioral economics theory relies on a unitary, expectancy-based view of motivation; however, research on self-determination theory differentiates between different types of motivation ranging from purely intrinsic motivation, which is the inherent satisfaction of engaging in an activity, to autonomous and internalized forms of extrinsic motivation, which is the desire to perform a behavior not for its own sake but because of its strong alignment with one’s closely held values and goals, to completely non-autonomous, externally-regulated extrinsic motivation, in which a behavior is performed solely to satisfy an external demand or obtain a contingent reward (e.g., a financial incentive). Meta-analytic research on these different types of motivation indicates that intrinsic motivation can be undermined by the imposition of external rewards if they are not structured properly [47]. Furthermore, while the use of financial incentives can increase the *quantity* of targeted behaviors, their effect on the *quality* of targeted behaviors is much more modest, particularly compared to the strong effects of internalized motivation on the quality of behavioral performance [48] [49]. This research demonstrates the importance of carefully structuring incentives and examining both their positive and potentially iatrogenic effects on providers’ behavior. This research also highlights the importance of using measures that assess CBT fidelity (i.e., quality) as opposed to CBT quantity when testing the effects of financial incentives on provider behavior.

Second, a few therapists reported that youth participation in the study may have increased client engagement and attendance to sessions because they were paid $10. This suggests that future studies testing incentives targeted at clients to increase engagement may be warranted [14]. However, the sample of clients obtained for this study were highly experienced in therapy, having on average attended 10 sessions, compared to the national modal number of sessions (i.e., 1 session) [50]. Thus, the current study provides little information about engagement and retention in an EBP. The future study should include a balanced sample of new and experienced clients. Third, with regard to the social incentives, we received feedback from leaders indicating that the condition may not have been robust enough to influence therapist motivation. Specifically, the leaders noted that the language drafted by the research team was not authentic and did not carry as much weight in one agency because they were already engaging in frequent public recognition. These comments will be taken into careful consideration when planning the social incentive condition in the larger trial to increase the saliency of the incentive. Studies to date on non-financial incentives for healthcare providers have primarily used either peer comparisons (showing providers how their performance compares to peers, [51]) or public reporting of provider or health system performance (for example, [52, 53]). Social incentives have been widely tested in other industries and settings to enhance performance and productivity. For example, in a study located at a Korea broadband internet firm, specific comments made to employees about positive behaviors and results was as successful as financial incentives in promoting performance [54]. Within the healthcare domain, it has been most commonly tested for community health workers in low- and middle-income settings, where it typically takes the form of a written or verbal recognition from a superior for outstanding performance or improvement (e.g., [55]). Our study is therefore among the first to use public recognition (vs. peer comparison or public reporting) as a social incentive in a mental health setting in a high-income country.

Additionally, the results of this study suggest the importance of assessing organizational context prior to selecting and evaluating implementation strategies [56]. Organizational characteristics that may influence the study results include the extent to which social incentives are already used by the agencies, the degree and quality of clinical supervision (which will shape therapists’ perceptions of the usefulness of feedback), the extent to which agencies engender proficient cultures that prioritize improvement in client well-being and clinician competence in up-to-date treatment practices, and the degree of innovation-values fit between CBT and agency leadership’s preferred theoretical orientation. We were surprised to learn that the feedback report was such a motivating feature of the study (reportedly potentially more motivating than the financial incentive). Given the power of audit and feedback to change behavior [57] and the lack of opportunities for therapists in the community to receive feedback on their session behavior, we have elected to include these reports in our future study as a stand-alone condition.

As a pilot, the study was not powered to detect the impact of the implementation strategies on changes in CBT adherence over the course of study [58]. However, in exploratory analyses, we found a trend suggesting that the financial incentive condition may have had a stronger effect on CBT adherence compared to the social incentive condition. If replicated in the larger study, this corroborates previous work that suggests that financial incentives increase implementation of an EBP for substance use disorders in youth [23]. Our preliminary results suggest that it may be possible to change clinicians’ EBP implementation behavior using incentives. One consideration based upon the pattern of results observed is the duration of the active intervention. In our study, the duration of incentive-oriented implementation strategies was brief (i.e., 4 weeks). Initially, there was high adherence to CBT which then decreased over the course of the 4 weeks. One possible explanation for this finding is that changes in initial behavior may have been due to observation (i.e., the Hawthorne effect). Given the trend of decreasing adherence over this brief study, future research which understands the long-term effects of incentive-oriented strategies is critical. To put the adherence rates into context, observations of therapist practices in usual care settings indicate that when therapists use EBPs, average therapist adherence is of low intensity (i.e., equivalent to a score of 2 or 3 on the TPOCS-RS ([35, 59, 60]). Therapist CBT adherence in this sample varied across CBT interventions; however, the average maximum score across the sample on the TPOCS-RS suggested that most therapists delivered CBT at least at a moderate intensity across the recorded sessions, which is promising.

Conducting this pilot work has been informative in the design of a larger cluster randomized trial in which we plan to test the comparative effectiveness of two incentive-based implementation strategies compared to implementation-as-usual to increase clinician use of CBT; this trial will be submitted to the National Institute of Mental Health. If funded, our plan is to randomize agencies implementing CBT in Philadelphia public mental health agencies to one of three implementation strategies: (1) implementation-as-usual (IAU): therapists receive written performance feedback only as consistent with the EBP initiatives approach in the city of Philadelphia; (2) financial incentive: weekly financial payment, plus feedback; (3) social incentive: public ranking on a weekly “leaderboard” listing all enrolled therapists, plus feedback. Randomization will occur at the agency level in order to address contamination concerns as well as leader and ethical concerns regarding the importance of giving all therapists within a single agency the opportunity to earn extra compensation. Therapists will receive rewards in conditions 2 and 3 if they provide CBT at a prescribed fidelity criterion. Outcomes will include adherence to CBT and the costs and cost-effectiveness of the three implementation strategies. Including the costs and cost-effectiveness aspect of this work is important to inform the scalability of such an approach. The design of this trial was directly informed by our experiences from this pilot study and includes randomization at the organization rather than clinician level and a stronger social incentive condition that is more directly driven by principles of behavioral economics [26].

Based on our experience and results from this pilot study, we will also measure and test agency-level factors that may moderate the effects of incentives on clinicians’ CBT fidelity. Innovation-values fit [61] is an important potential agency-level moderator given Philadelphia’s long history as a home for evidence-based family therapy approaches and the possibility that agencies and their leadership may be more aligned with these approaches compared to CBT. Other important potential moderators include proficient organizational culture, which encompasses norms and behavioral expectations that clinicians prioritize improvement in client well-being and exhibit competence in up-to-date treatment practices, molar organizational climate, which describes clinicians’ shared perceptions of the extent to which the work environment supports their personal well-being and the extent to which agencies currently use social incentives [62]. Both proficient culture and molar climate have been linked to improved implementation of evidence-based practices in behavioral health services ([5, 63–65]). As a result, we expect that incentives may not have as powerful an effect on clinicians’ behaviors in agencies with these positive organizational characteristics. Conversely, proficient organizational cultures and supportive climates may increase the effects of incentives, particularly social incentives, by framing them as a consistent part of an overall organizational priority for improving client well-being. Positive innovation-values fit, in which CBT is concordant with leaders’ and clinicians’ preferred approaches to treatment, should also enhance the effects of incentives on therapists’ practice behavior.

### Implications for implementation science

This pilot study has a number of implications for the forward movement of the field of implementation science. First, the findings demonstrate the utility of conducting iterative pilot work. Important insights were gleaned using mixed-methods as part of the pilot that have informed the larger trial design and demonstrate the importance of conducting pilot work prior to launching a fully powered trial. Second, this pilot work was engendered by observational work ([5, 10–12]) suggesting the importance of tailored implementation strategies that address lack of motivation based on the behavior change components of reward and threat [20]. Third, this pilot work uses theory to delineate the targets and mechanisms of the developed implementation strategies, which moves the field towards building causal theory [66]. In the trial to ensue, we will test the comparative effectiveness of these implementation strategies while also measuring mechanisms, which will also move the field in this direction.

## Conclusions

The aim of this pilot study was to test the feasibility and acceptability of two incentive-based implementation strategies, as well as preliminarily examine therapist adherence to CBT. We were able to feasibly administer the implementation strategies, and semi-structured interviews indicate good acceptability. The findings underscore the importance of conducting iterative pilot work to inform the design of future trials.

## Additional file

Additional file 1: Email from leaders to therapists. (DOCX 21 kb)

## Abbreviations

CBT: Cognitive-behavioral therapy
EBP: Evidence-based practice
FI: Financial incentive
IAU: Implementation-as-usual
SI: Social incentive
TF-CBT: Trauma-focused cognitive-behavioral therapy
TPOCS-RS: Therapy Process Observational Coding System for Child Psychotherapy-Revised Strategies Scale
