# Supplementary material for: Feasibility and acceptability of two incentive-based implementation strategies for mental health therapists implementing cognitive-behavioral therapy: a pilot study to inform a randomized controlled trial
Source: Implement Sci. 2017 Dec 15;12:148. doi: 10.1186/s13012-017-0684-7 (PMC5732393; doi:10.1186/s13012-017-0684-7)
Supplement: Additional file 1: — Email from leaders to therapists. (DOCX 21 kb) [file 13012_2017_684_MOESM1_ESM.docx]

Additional file 1. Email from leaders to therapists

Dear Team,

Researchers from the University of Pennsylvania have been conducting a study at our agency for the past few weeks. Please join me in congratulating therapists, XXX and XXX, on their extensive use of CBT strategies, an evidence-based practice, in a randomly selected session that was coded by the research team. I am very proud of the dedication, commitment, and use of evidence-based strategies displayed by our therapists. Please join me in celebrating XXX and XXX’s accomplishments by congratulating them on their hard work.

All the best,

XXX
